# Phase Coupled Firing of Prefrontal Parvalbumin Interneuron With High Frequency Oscillations

**DOI:** 10.3389/fncel.2020.610741

**Published:** 2020-11-25

**Authors:** Yanting Yao, Mengmeng Wu, Lina Wang, Longnian Lin, Jiamin Xu

**Affiliations:** ^1^Shanghai Key Laboratory of Brain Functional Genomics (Ministry of Education), School of Life Sciences, East China Normal University, Shanghai, China; ^2^NYU–ECNU Institute of Brain and Cognitive Science, New York University Shanghai, Shanghai, China; ^3^Brain and Spinal Cord Clinical Center, Tongji University, Shanghai, China

**Keywords:** optogenetics, prefrontal cortex, parvalbumin interneurons, firing pattern, high frequency oscillation

## Abstract

The prefrontal cortex (PFC) plays a central role in executive functions and inhibitory control over many cognitive behaviors. Dynamic changes in local field potentials (LFPs), such as gamma oscillation, have been hypothesized to be important for attentive behaviors and modulated by local interneurons such as parvalbumin (PV) cells. However, the precise relationships between the firing patterns of PV interneurons and temporal dynamics of PFC activities remains elusive. In this study, by combining *in vivo* electrophysiological recordings with optogenetics, we investigated the activities of prefrontal PV interneurons and categorized them into three subtypes based on their distinct firing rates under different behavioral states. Interestingly, all the three subtypes of interneurons showed strong phase-locked firing to cortical high frequency oscillations (HFOs), but not to theta or gamma oscillations, despite of behavior states. Moreover, we showed that sustained optogenetic stimulation (over a period of 10 s) of PV interneurons can consequently modulate the activities of local pyramidal neurons. Interestingly, such optogenetic manipulations only showed moderate effects on LFPs in the PFC. We conclude that prefrontal PV interneurons are consist of several subclasses of cells with distinct state-dependent modulation of firing rates, selectively coupled to HFOs.

## Introduction

The prefrontal cortex (PFC) is a critical region responsible for higher cognitive functions including decision making, value estimation, attention, social cognition, working memory and motor control (Miller, 2000). The excitation/inhibition (E/I) balance of cortical circuits plays an important role in information processing during cognitive behaviors. Moreover, E/I balance has been suggested to contribute to the modulation of neural network oscillations (Atallah and Scanziani, 2009; Xue et al., 2014). Local field potential oscillations in the neocortex of mammalians, such as delta (1–4 Hz), theta (4–8 Hz), gamma (30–80 Hz), and the recently reported high-frequency oscillations (HFOs > 100 Hz), are related to various cognitive processes (Ward, 2003). Theta oscillations have been observed in the PFC during spatial memory, working memory and attention tasks (Tamura et al., 2017). Theta-associated gamma rhythms are considered to be involved in cognitive processes including working memory, sensory and visual responses (Cardin et al., 2009; Tamura et al., 2017). HFOs have been observed when the cortex received excitatory inputs (Engel et al., 2009). But the mechanism of HFO generation and their function in cortical information processing is still elusive.

Inhibitory inputs provided by GABAergic interneurons are essential for optimizing the E/I ratio. The loss of PFC GABAergic inhibitory inputs will lead to an elevation in the E/I ratio of pyramidal neurons, which is considered to be one etiology of cognitive impairments such as schizophrenia and autism (Yizhar et al., 2011). As one of the main sources of inhibitory inputs, PV-positive GABAergic interneurons constitute about 40% of the total cortical interneurons (Tremblay et al., 2016). Previous studies have shown that PV interneurons provide powerful inhibitory innervations onto postsynaptic pyramidal neurons (Pfeffer et al., 2013). They are also involved in regulating a variety of cognitive behaviors (Kim et al., 2016). Electrophysiological recordings in brain slices have stated that PV interneurons in medial prefrontal cortex (mPFC) mediate the feedforward inhibition circuits, which is crucial for maintaining cortical E/I balance (Lee et al., 2014). Moreover, neuronal firing activity of some PFC PV interneurons have been reported to be phase-locked to gamma oscillations (Kim et al., 2016). But still, little is known toward the *in vivo* firing patterns of cortical PV interneurons across behavioral states. We set to investigate whether and how PV interneurons may participate in regulating LFP dynamics, including gamma oscillation, under distinct behavioral states.

To answer the above two questions, we performed *in vivo* electrophysiological recording in the mPFC region of free moving mice across various behavioral states, including active wakefulness (AW), quiet wakefulness (QW), rapid-eye-movement (REM) sleep and slow-wave sleep (SWS) states. With the help of optogenetic tools, we described the *in vivo* firing patterns of PFC PV interneurons, and investigated their relationship with LFP oscillations. We further examined the effects of optogenetic manipulations of PV cells on E/I balances and local neural network dynamics.

## Materials and Methods

### Animals

All experiment procedures were carried out in accordance with protocols approved by the Laboratory Animal Management Committee at East China Normal University (Huashishe [2014] No. 6). Two mouse lines [PV-IRES-Cre, Jax No. 008069 (Hippenmeyer et al., 2005) and Ai32 Jax No. 012569 (Madisen et al., 2012)] were used to generate PV-ChR2-EYFP transgenic mice. All animals are offered with 12 h alternating day and night illumination and free access to food and water.

### Animal Behavioral State Assessment

We choose four basic behavioral states for further analysis: active wakefulness (AW), quiet wakefulness (QW), rapid-eye-movement (REM) sleep, and slow-wave sleep (SWS). The animal behavioral states were identified through videos recorded via a camera over the recording arena, assisted by simultaneously recorded LFP signals from the PFC and hippocampal dCA1. The AW state was the periods when mice performed proactive physical movements. The appearance of sleeping posture and cortical sleep spindles (7–12 Hz) marks sleep states. During sleep, the periods when slow waves (<1 Hz) and delta rhythms constantly existed in both recording sites (and with hippocampal sharp wave ripple events occurred occasionally) were identified as SWS state, while periods with continuous theta rhythms were classified as REM state. The QW state was defined as the periods when no obvious physical movement, sleeping posture, or sleep spindles in the filtered LFP signal could be detected.

### *In vivo* Electrophysiological Recordings

Microdrive electrodes of 64 or 96 channels were designed for recording across multiple brain regions. The microdrive foundation was adopted from our previous work (Lin et al., 2006). For 64-channel, a few (no more than 4) tetrodes were placed in a bundle targeting hippocampal dorsal CA1 (dCA1), and the others were placed in a bundle targeting mPFC. For 96-channel microdrive electrodes, 16 were placed in mPFC. and 8 tetrodes were placed in dCA1 to help characterize the basic behavioral states such as REM and SWS based on hippocampal LFP dynamics. The tetrode tips were trimmed and electroplated with plating liquid (24K gold, Promex Industries, United States) to reach a final impedance of 500–800 kΩ via an electrode impedance tester (IMP-I, Bak Electronics, United States). An optical fiber was inserted in the middle of the tetrode bundle (optrode), targeting the mPFC with an 0.5 mm indentation from the tetrode tips.

The mice used for recording were housed individually in a rectangular cage (470 mm length × 315 mm width × 260 mm height) with free access to water and food pellets, and handled for a week before surgery (30 min per day). A total number of nineteen PV-ChR2-EYFP mice (2–4 months old, ranging 22–28 grams prior to the implantation surgery, no preference on sex) were surgically implanted with microdrive electrode according to the protocols described in Lin et al. (2006). The tetrode bundles were implanted in unilateral mPFC (AP + 1.94 mm, ML + 0.50 mm, DV −1.50 to −1.90 mm, targeting layer 2 to layer 5 of the prelimbic area of mPFC) as well as the ipsilateral hippocampal dCA1(AP −2.30 mm, ML + 2.00 mm, DV −1.00 mm).

After a recovery of 4–7 days post the surgery, mice underwent electrophysiological observations. Electrophysiology signals were recorded by Plexon MAP system while animal movement was monitored via a video camera. The signals from electrodes were filtered through the preamplifiers (400–7,000 Hz for neuronal spikes, 0.7–300 Hz for LFP), and then sampled at 40 kHz (spikes) or 1 kHz (LFP). The electrode bundles were advanced at a rate of no more than 70 μm every 3 days. We started recording when the spikes of putative interneurons (narrow waveforms with a mean firing rate higher than 5 Hz) were detected in mPFC, and characteristic LFP signals of stratum pyramidale (i.e., sharp wave ripples, Buzsaki et al., 1992) were evident in dCA1.

### Optogenetic Stimulation

Blue laser stimulator (DPSS Laser, 470 nm, Inper, China) was used to activate neurons labeled with ChR2 in the PV-ChR2-EYFP mice. Unless otherwise specified, the laser power was set to optimum (usually 5–20 mW), with 5 ms pulse width at 1 Hz frequency for 100 trials for each neuron. Spikes fired within 10 ms after laser onset were considered to be light evoked spikes. We calculated the light triggered spiking probability by measuring the proportion of the number of trials in which at least one spike was triggered by light stimulation over the total number of trials. Neurons with a light-induced firing probability over 60% were identified as PV positive interneurons. For laser power test, laser power was set to 5, 10, 15, and 20 mW. For laser frequency test, stimulus frequency was set to 0.5, 1, 2, 10, 20, and 40 Hz. For sustained stimulation, the laser stimulation was continuously delivered for 10 s.

### Immunohistochemistry and Microscopy

After all recording experiments were completed, mice were deeply anesthetized with pentobarbital sodium (0.1 mg/g body weight) and perfused transcardially with 0.01 M PBS followed by 4% paraformaldehyde (w/v, in PBS). The brain was stripped out and underwent gradient dehydration in 20 and 30% sucrose solution (w/v, in PBS). Coronal sections (30 μm) were prepared with a freezing microtome (CM1520, Leica, United States). Sections were penetrated with 0.5% Triton X-100 (in PBS) at room temperature for half an hour, followed by goat serum (16210-064, Gibco, Life Technologies, Thermo Fisher Scientific, United States) at room temperature for 1 h. Sections were first incubated with primary antibodies (Rabbit IgG anti-PV, PV27, SWant, CH, 1:1,000; Mouse Monoclonal IgG anti-GFP, 600-301-215, Rockland, United States, 1:500) diluted in the antibody diluent solution (003118, Life technologies) overnight at 4°C. Following a 3 × 10 min PBST washing, the sections were then incubated with second antibody (Alexa Fluor 594 Goat anti Rabbit IgG, A-11012, Life Technologies, Thermo Fisher Scientific, United States, 1:500; Alexa Fluor 488 Goat anti Mouse IgG, A-11001, Life Technologies, Thermo Fisher Scientific, United States, 1:500) for 2 h and DAPI (C1006, Beyotime, CN, 1:1,000) for 10 min at room temperature. Following another 3 × 10 min PBST washing, the sections were mounted with 30% glycerol and characterized by confocal microscopy (TCS SP8, Leica, United States).

### Data Processing

Spike sorting was performed with Offline Sorter 2.0 software (Plexon, Dallas, TX) as previously described (Zhang et al., 2012). Spikes of single units were converted into ^∗^.nex files together with original LFP signals for further processing with MATLAB.

The mPFC LFP signals were band-pass filtered in the delta (2–5 Hz), theta (4–8 Hz), slow gamma (30–50 Hz), fast gamma (50–80 Hz) and high-frequency (100–250 Hz) bands using elliptic filter. To detect HFO events, the root mean square of the filtered signal was calculated by sliding a 10 ms window every 1 ms. Epochs with 2 standard deviations above the background mean power were designated as HFO episodes. Then the time window was moved forward and backward to detect the beginning and the end of each HFO episode, the threshold was set to 1 standard deviation above the background mean power. In addition, the dCA1 LFP signals were band-pass filtered in the delta (2–4 Hz), theta (4–12 Hz), gamma (30–80 Hz) and ripple (100–250 Hz) bands using elliptic filter. To detect ripple events, the root mean square of the filtered ripple signal was calculated by sliding a 10 ms window every 1 ms. Epochs with 5 standard deviations above the background mean power were designated as ripple events. Then the time window was moved forward and backward to detect the beginning and the end of each ripple episode, the threshold was set to 2 standard deviations above the background mean power.

### Power Spectrum Analysis

Welch Method were employed for LFP power spectral density (PSD) analysis, with 512 points fast Fourier transform (FFT), 512-ms 1/4 overlapping Hanning window. The power spectrograms in Figures 3–5 were conducted based on adaptive autoregressive (AAR) model and Kalman filtering (Arnold et al., 1998). Define *X*_*t*_ as the time sequence of LFP, the AAR model of the order *p* of *X*_*t*_ can be expressed as:

$$X_{t}=\sum_{k=1}^{\text{p}}A_{t}^{\left(k\right)}\,X_{t-k}+E_{t}$$

in which *t* is time, $A_{t}^{\left(k\right)}$ is the parameter. E_*t*_ is zero mean Gaussian noise process whose variance is *Σ*_*t*_. Given the state vector $A_{t}={\left(A_{t}^{\left(1\right)},A_{t}^{\left(2\right)},\ldots ,A_{t}^{\left(p\right)}\right)}^{T}$, and the observed variable H_*t*_ = (*X*_*t*−1_,*X*_*t*−2_,…,*X*_*t*−*p*_)*T*, T represents matrix transpose. Then *X*_*t*_ can be expressed as:

$$X_{t}=H_{t}^{T}\,A_{t}+E_{t}.$$

The change of state can be described by random walk model *A*_*t* + 1_ = A*t* + *W*_*t*_, here E*t* and W*t* are uncorrelated zero mean Gaussian noise processes, whose variances are $V_{e_{t}}=\sigma_{e_{t}}^{2}$ and $V_{{\text{w}}_{t}}=\sigma_{w_{t}}^{2}$ separately, Then the first step forecast is:

$${\hat{A}}_{t|t-1}=E\,\left[A_{t}|X_{0},X_{1},\ldots ,X_{t-1}\right].$$

Here, Kalman filtering is introduced for parameter estimation. The Kalman filtering equations are as follows (Andreassen et al., 1979):

$${\hat{A}}_{t|t-1}=\left(I-K_{t}\,H_{t}^{T}\right)\,{\hat{A}}_{t|t-1}+K_{t}\,X_{t},$$

$${\text{K}}_{t}=\sum_{t|t-1}H_{t}\,{\left(H_{t}^{T}\,\sum_{t|t-1}H_{t}+\sigma_{e_{t}}\right)}^{-1},$$

$$\sum_{t+1|t}\Delta \,E\,\left[\left(A_{t+1}-{\hat{A}}_{t+1|t}\right)\,\left(A_{t+1}-{\hat{A}}_{t+1|t}\right)|X_{t}\right]=$$

$$\sum_{t|t-1}-K_{t}\,H_{t}^{T}\,\sum_{t|t-1}+\sigma_{\omega_{t}},$$

$${\hat{A}}_{t|t}={\hat{\text{A}}}_{t|t-1}+K_{t}\,\left(X_{t}-H_{t}^{T}\,{\hat{A}}_{t|t-1}\right),$$

$$\sum_{t|t}=\sum_{t|t-1}-K_{t}\,H_{t}^{T}\,\sum_{t|t-1},$$

in which *I* is identity matrix, K*t* is Kalman gain matrix.

Then the state vector *A*_*t*_ can be estimated as:

$${\hat{A}}_{t|N}=E\,\left[A_{t}|X_{0},X_{1},\ldots ,X_{N}\right],0\leq t\leq N,$$

a fixed-interval smoothing procedure was applied to avoid the time-lag in the estimates (Tarvainen et al., 2004). And the real-time PSD of signal *X*_*t*_ at frequency *ω* can be given by:

$$f\,\left(t,\omega \right)=\frac{\sigma_{e}^{2}\,\left(t\right)}{f_{s}\,{\left|1-\sum_{j=1}^{p}A_{t}^{\left(j\right)}\,e^{-2\,i\,\pi \,\omega \,j/f_{s}}\right|}^{2}},$$

where f_s_ is the sampling frequency.

### Phase-Locking Analysis

Phase-locking analysis was performed according to a previous study (Siapas et al., 2005). The amplitude *A*(*t*) and phase ϕ(*t*) of LFP signals were extracted by applying the Hilbert transform. Phase of spike trains at times *S* = {τ_*k*_|*k* ∈ {1,2,…,*n*}} is then given by ϕ_*s*_ = {ϕ(*t*)|*t* ∈ *S*}. To evaluate the presence of phase-locking, Rayleigh test for circular uniformity was performed. Briefly, the preferred phase μ is given by the mean direction of ϕ_*s*_. $\bar{R}$ is the mean resultant length of unit vector ϕ_*s*_. The Rayleigh statistic is $Z=n\,{\bar{R}}^{2}$. And the probability for the presence of phase-locking is given by

$$P=e^{-Z}\,\left(1+\frac{2\,Z-Z^{2}}{4\,n}-\frac{24\,Z-132\,Z^{2}+76\,Z^{3}-9\,Z^{4}}{288\,n^{2}}\right).$$

Here we take *P* < 0.001 as significant phase-locking. The phase of spike trains was fit with a von Mises distribution with density

$$f\left(\phi \right)=\frac{1}{2\,\pi \,I_{0}\,\left(\kappa \right)}e^{\kappa \,\cos\left(\phi -\mu \right)},\left(-\pi \leq \phi <\pi ,0\leq \kappa <\infty \right).$$

The concentration parameter κ can be solved from the equation $I_{1}\,\left(\kappa \right)/I_{0}\,\left(\kappa \right)=\bar{R}$ by applying a numerical zero finding routine. *I*_*i*_(*x*) is the modified Bessel function of order *i*.

### Pairwise Phase Consistency

Pairwise phase consistency (PPC) is a bias-free measurement of the phase synchronization of neuronal spiking in relation to LFP (Vinck et al., 2010, 2012). For a given frequency *f*, the PPC of a spike train is defined as:

$$P\,P\,C=\frac{\sum_{j=1}^{N}\sum_{k\neq j}^{N}\left(\sin\theta_{j}\,\sin\theta_{k}+\cos\theta_{j}\,\cos\theta_{k}\right)}{N\,\left(N-1\right)},$$

in which θ_*j*_ and θ_*k*_ denote phase of the *j*-th and *k*-th spike at frequency *f*, *N* denotes the total number of spikes.

## Results

### Cell-Type-Specific Labeling and Activation of PV Interneurons in the PFC

**FIGURE 1:**
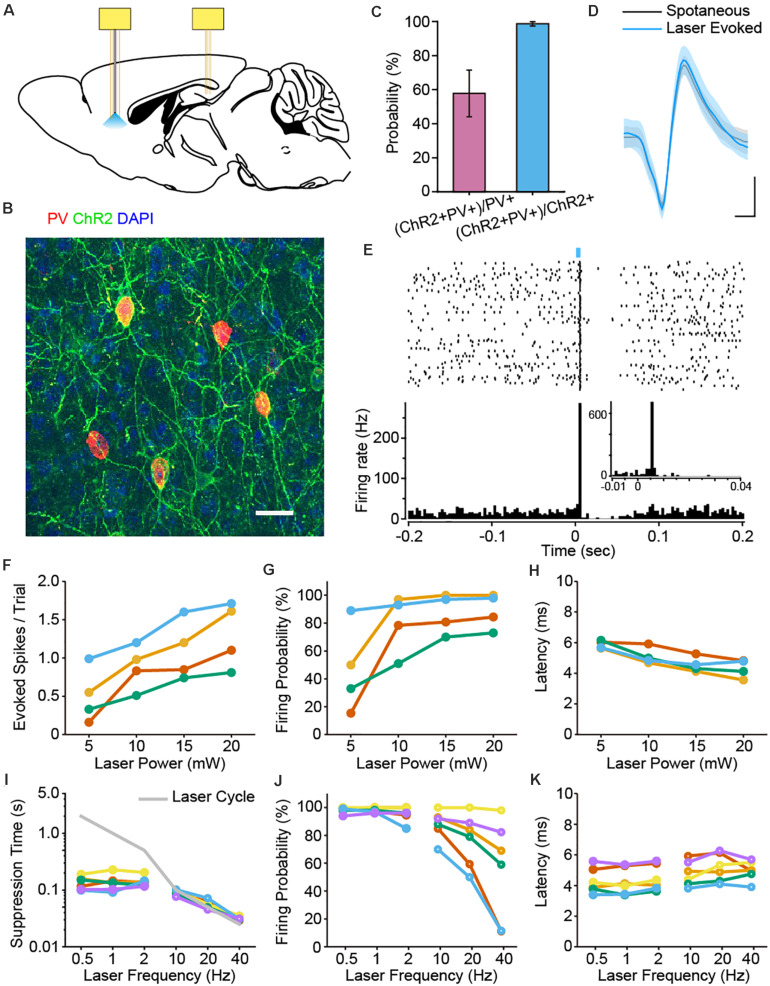
Optogenetic identification of PFC PV interneurons and characterization of laser stimulation parameters. **(A)** Schematic diagram of the placement of two electrode bundles: an optrode targeting the PFC and a tetrode bundle in the dCA1 of the hippocampus. **(B)** Immunostaining of PV (red), EYFP (green) and DAPI (blue) confirmed the co-localization of PV and ChR2 in the PFC of PV-ChR2-EYFP transgenic mice. Scale: 50 μm. **(C)** Expression efficiency statistics revealed that 98.63% ± 1.28% of the ChR2 + neurons were PV+, whereas 57.82% ± 13.67% of the labeled PV neurons were ChR2 + (*n* = 7 mice). **(D)** Example spike waveforms from one PV neuron. The waveforms of the spontaneous (black) and laser-evoked spikes (blue) were almost identical (mean ± SD of 100 spikes, Pearson Correlation, *R* = 0.998, *p* = 1.27e-62). Scale: 0.2 ms, 0.2 mV. **(E)** Peri-stimulus raster and histogram of an example PV interneuron upon laser pulse stimulation (1 Hz, 5 ms pulse, 100 trials, bin = 3 ms). Note the brief suppression response right after the laser evoked firing of PV neuron. Enlarged view shows light-induced spike latency around 6 ms (1 Hz, 5 ms pulse, 100 trials, bin = 1 ms). **(F–H)** The impact of laser power on the number of evoked spikes per trial **(F)**, firing probability **(G)** and spike latency **(H)** for PV interneurons. Each color corresponds to a single neuron, the same below (*n* = 4 neurons). Neuronal firing responses increased with higher laser stimulation power. **(I–K)** The impact of laser frequency on suppression time **(I)**, firing probability **(J)** and latency **(K)** for PV interneuron activation. Solid circles, lower frequency; open circles, higher frequency (*n* = 6 neurons). Neuronal firing probability decreased at higher stimulation frequencies **(J)**.

In order to study the *in vivo* firing properties of PV interneurons, we need to first identify the PV interneurons in the PFC in freely moving mice. Optogenetic tagging of neuronal firing has been proven to be practical for such identification. Tetrode arrays together with an optical fiber (optrode) were stereotaxically implanted into mPFC in PV-ChR2-EYFP double transgenic mice (Figure 1A), which were produced by crossing a PV-IRES-Cre line (Jax No. 008069) (Hippenmeyer et al., 2005) with the Ai32 ChR2/EYFP line (Jax No. 012569) (Madisen et al., 2012).

We first examined the co-expression profile of PV and ChR2 in the PFC. We found that 98.63% ± 1.28% of the neurons labeled with ChR2 were positive with PV antibody, while 57.82% ± 13.67% of the PV + neurons co-expressed ChR2 (Figures 1B,C).

We then applied laser pulse stimulations via the optical fiber in mPFC during electrophysiological recordings. Indeed, some neurons exhibited stable light evoked spiking responses upon 470 nm laser stimulation. Importantly, the waveforms of spontaneous and optogenetically evoked spikes are almost identical, confirming these cells as PV interneurons (Figure 1D). Laser pulse stimulation induced spiking activities of PV neurons, followed by a brief suppression of firing activities right after the light evoked responses (1 Hz, 5 ms laser pulse, Figure 1E). Neurons with a light-induced firing probability over 60% were identified as PV positive interneurons. Under this criterion, we identified a total number of 18 PV interneurons in 10 mice.

To better understand the impact of laser stimulation on the firing activities of PV interneurons, we tested various laser stimulation parameters. First, we tested the effect of laser power on spike responses of 4 PV interneurons. As expected, PV interneurons were more effectively activated with higher laser power (Figure 1F). Some even fired more than one spike within a single trial, which led to a saturation of the firing probability at higher laser power (Figure 1G). We next measured the latency of light evoked response to laser power, which is defined as the duration from the laser onset to the first evoked spike. We found that response latency slightly decreased as the light stimulation power increased (Figure 1H). This is consistent with previous study showing that higher light intensity benefits the rapid activation of ChR2 (Ishizuka et al., 2006).

We also tested the effects of laser stimulation frequency (0.5–40 Hz) on the firing responses of PV interneurons. When PV interneurons were stimulated at a lower frequency (i.e., 0.5, 1, and 2 Hz), the cycle of laser stimulation was much longer than the intrinsic suppression time of PV interneurons. As such, the corresponding firing probabilities were able to remain at a higher level (Figure 1J). In contrast, when PV interneurons were stimulated at a higher frequency (i.e., 10, 20 or 40 Hz), the suppression-phase time-duration of the laser-triggered suppression responses were artificially shortened and masked by the rapid laser cycles (Figure 1I). This came at a cost of the dramatic decline in firing probability (Figure 1J), whereas the latency in PV cells did not show such drastic changes (Figure 1K).

### Behavioral State-Dependent Firing Patterns of PFC PV Interneurons

**FIGURE 2:**
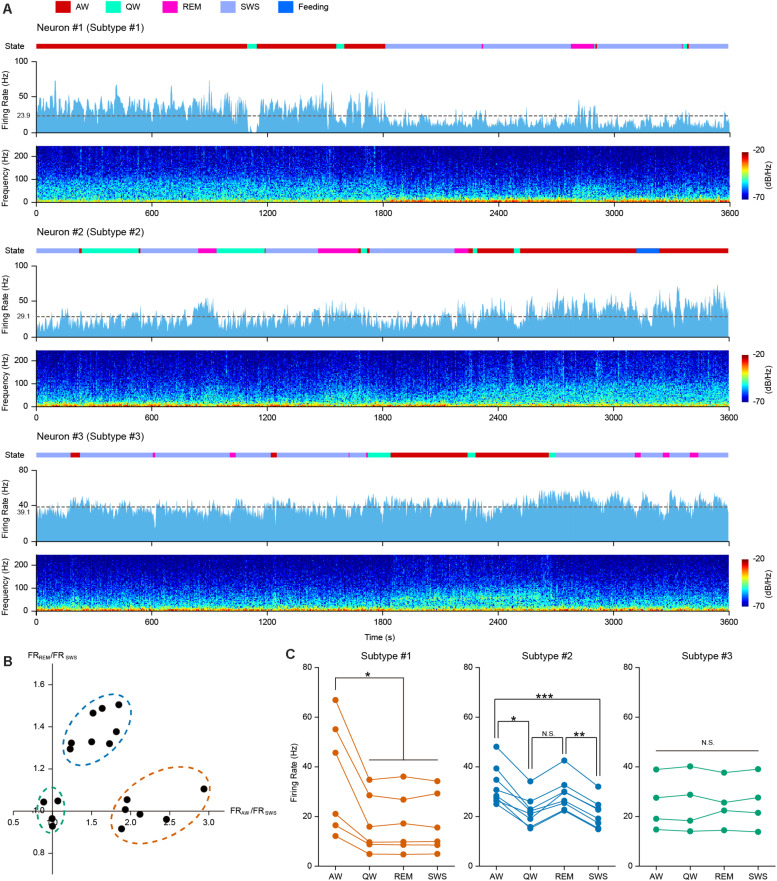
PFC PV interneurons exhibited behavior state-dependent firing patterns. **(A)** One hour continuous monitoring of the firing activities of 3 example PFC PV interneurons. Each example neuron represents one interneuron subtype, showing behavior state-dependent firing patterns. Different behavioral states were associated with changes in neuronal firing rates and LFP power spectrograms. Top row, color coded behavior states (color scheme at the top of the figure); middle row, neuronal firing rate histogram (bin = 2 s) and mean firing rate (dashed gray line, average firing rate on the left); bottom row, LFP power spectrogram. **(B)** Relationship between FR_*AW*_/FR_*SWS*_ and FR_*REM*_/FR_*SWS*_, each dot represents one opto-tagged PV interneuron. 18 PV neurons were clustered into three subtypes (shown in dashed circles. orange, subtype #1; blue, subtype #2; green, subtype #3). **(C)** Mean firing rate of all the 18 recorded PV interneurons under different behavior states, clustered into 3 subtypes (color coded in orange, blue and green, *n* = 6, 8, and 4 neurons in each subtype). Each line connecting 4 dots represents one single neuron. Note the difference in behavior state-dependent firing rate change of each interneuron subtype (Friedman Test, *p*_*subtype #*__1_ = 0.013, *p*_*subtype #*__2_ = 9.80e-5, *p*_*subtype #*__3_ = 0.96; Dunn-Bonferroni *post hoc*, *p < 0.05, **p < 0.01, ***p < 0.001, N.S., not significant).

Neuronal activities are often associated with different behavioral and cognitive states. We carried out long-period continuous recordings for the 18 opto-tagged PFC PV interneurons to investigate temporal firing dynamics across behavior states. Neuronal firing activity of 3 example PV neurons over 1 h recording across different behavioral states was illustrated in Figure 2 (light blue histogram), along with spectrograms for the simultaneously recorded LFP at the bottom of each example neurons. Based on neuronal firing kinetics during the four basic behavioral states, we categorized PV interneurons into three subtypes. The first subtype (example Neuron #1 in Figure 2A) exhibited a higher firing rate during AW state over other states (Figure 2C, left). The second subtype of PV cells (example Neuron #2 in Figure 2A) tended to fire in a higher rate during both AW and REM states (Figure 2C, middle). The third subtype of PV interneurons (example Neuron #3 in Figure 2A) did not show significant firing rate changes across all behavior states (Figure 2C, right).

To quantitatively measure these differences, we compared the mean firing rates during AW and REM states against that during SWS state for each opto-tagged PV interneuron. FR_*AW*_/FR_*SWS*_ versus FR_*REM*_/FR_*SWS*_ of each PV neuron were plotted, where FR stands for the mean firing rate of each state (Figure 2B). The 18 PV neurons could be evidently clustered into three subtypes according to their behavior state-dependent firing properties.

PV interneuron subtype #1 has 6 neurons (orange circle in Figure 2B), showing high firing rates during AW state. The firing rate during AW state was significantly higher than that during the other three states. Interestingly, the firing rates during SWS, REM and QW states were consistently lower (Figure 2C, left panel. Friedman Test, *n* = 6 neurons, *p* = 0.013; Dunn-Bonferroni *post hoc*, *p*_*AW*__–__*QW*_ = 0.044, *p*_*AW*__–__*REM*_ = 0.044, *p*_*AW*__–__*SWS*_ = 0.044). Therefore, we speculate that subtype #1 PV cells may be associated with the modulation of active behaviors. Subtype #2 PV cells exhibited higher firing rates during AW and REM states. The FR_*AW*_/FR_*SWS*_ and the FR_*REM*_/FR_*SWS*_ ratio plot indicated that 8 neurons fall into the class of subtype #2 (blue circle in Figure 2B). The mean firing rate plot in Figure 2C showed higher firing rates of subtype #2 neurons during both AW and REM states in comparison to QW and SWS states (Figure 2C, middle panel. Friedman Test, *n* = 8 neurons, *p* = 9.8e-5; Dunn-Bonferroni *post hoc*, *p*_*AW*__–__*QW*_ = 0.022, *p*_*AW*__–__*SWS*_ < 0.001, *p*_*REM*__–__*SWS*_ = 0.006). The remaining four PV interneurons belonged to subtype #3 (green circle in Figure 2B), showing comparable firing rates across all four behavior states (Figure 2C, right panel. Friedman Test, *n* = 4 neurons, *p* = 0.96).

Taken together, these results suggested that PV cells in the PFC likely reflect multiple distinct groups of interneurons. Some of these subtypes clearly exhibited behavior state-dependent firing rate modulation.

### Phase Coupling of PV Cells With High-Frequency Oscillations During AW State

**FIGURE 3:**
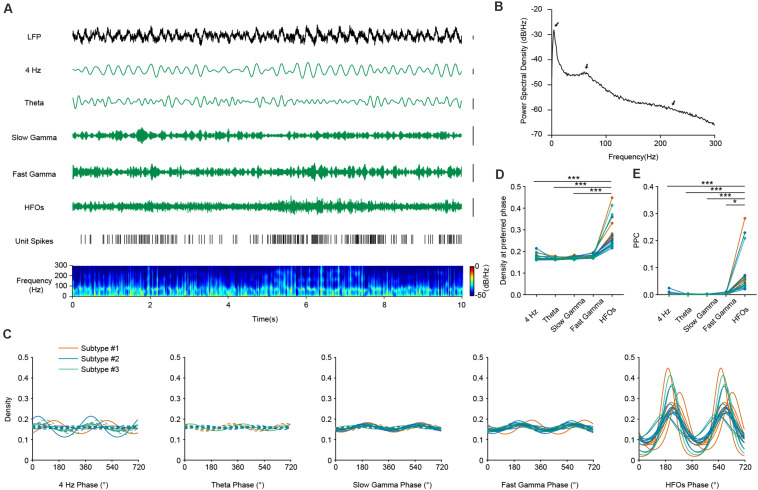
The firing phase-coupling of PFC PV interneurons to LFP HFOs during AW state. **(A)** Example traces showing PV interneuron spikes and different LFP oscillation components during AW state. From top to bottom, LFP signal recorded in the PFC, cortical 4 Hz oscillation, theta oscillation, slow and fast gamma oscillation, HFOs filtered from LFP, PV interneuron spiking activity (each stick represents one action potential fired by the neuron), and power spectrogram of LFP (10 s of data is shown, scale, 0.1 mV). Note the co-activity of elevated neuronal firing and increased power of HFO components in the power spectrogram (e.g., from 5 to 8 s). **(B)** Power spectral density during AW state of one example recording. Theta, gamma and HFO components exhibited prominent power (marked by arrows). **(C)** Firing phase distributions of 3 subtypes of PV interneurons with different oscillation components. Significant phase-locking is shown with solid lines, and non-significant phase-locking shown with dashed lines (Rayleigh’s test, *p* < 0.001 as significant phase-locking). Each colored line represents one single PV neuron (*n* = 18 neurons). PV interneurons fire significantly phase-locked to HFOs during AW state, compared to that with other oscillations. **(D)** Peak phase coupling density with different oscillations at preferred phase of PV interneuron spikes during AW states (Friedman Test, *p* = 4.19e-10, Dunn-Bonferroni *post hoc*, ***, *p*_4 *Hz*__–__*HFO*__*s*_ < 0.001, *p*_*theta*__–__*HFOs*_ < 0.001, *p*_*slow gamma*__–__*HFOs*_ < 0.001, *n* = 18 neurons). **(E)** Comparison of pairwise phase consistency (PPC) analysis. Note that the phasic firing of PV interneurons to HFOs was much more predominant than that to other oscillations during AW state (Friedman Test, *p* = 7.77e-9, Dunn-Bonferroni *post hoc*, ***, *p*_4 *Hz*__–__*HFO*__*s*_ < 0.001, *p*_*theta*__–__*HFOs*_ < 0.001, *p*_*slow gamma*__–__*HFOs*_ < 0.001, *, *p*_*fast gamma*__–__*HFOs*_ = 0.016, *n* = 18 neurons).

To evaluate the relationship of the temporal dynamics of PV neuronal firing with LFP, we examined whether and how the activities of PV interneurons were associated with different oscillation components of the LFP, namely theta (4–8 Hz), slow and fast gamma (30–50 and 50–80 Hz), and high frequency oscillations and a recently reported 4 Hz oscillation component (Fujisawa and Buzsaki, 2011; Karalis et al., 2016; Figure 3A). Theta oscillations occur frequently during AW state in the mouse PFC, constantly accompanied by gamma and high-frequency rhythms (Figure 3B). We performed firing phase analysis between PV interneuronal firing and different components of LFP oscillations. We found 5 PV interneurons with significant phase-locked firing with 4 Hz oscillation (Figure 3C, left panel). Previous reports have shown that the firing activities of some hippocampal PV interneurons were highly correlated with hippocampal theta rhythms (Klausberger et al., 2003). Unlike the situation in the hippocampus, we found that most of the PFC PV cells (17/18) did not show significant phase-locked firing to theta oscillations (Figure 3C, second left panel). Among the 18 recorded PV neurons, 7 of them showed significant phase-locked firing to slow gamma oscillation, and 11 of them showed significant phase-locked firing to fast gamma oscillation (Figures 3C, middle panel and second right panel). Interestingly, we found that the activity of PV interneurons showed a strong correlation with LFP HFOs. They increased their firing rate when the power of HFO increased (Figure 3A). All the recorded PV interneurons were significantly phase-locked to the trough of HFOs during AW state (mean preferred phase 221.91°± 23.61°, Figure 3C, right panel). The phase-locked firing of PV interneurons with HFOs were much stronger than that with other oscillations (Figure 3D, Friedman Test, *p* = 4.19e-10, Dunn-Bonferroni *post hoc*, *p*_4Hz__–__*HFO*__*s*_ < 0.001, *p*_*theta*__–__*HFOs*_ < 0.001, *p*_*slow gamma*__–__*HFOs*_ < 0.001, *n* = 18 neurons).

To further quantify the strength of phase-locked firing of PFC PV interneurons with HFOs, we measured pairwise phase-consistency (PPC) of each recorded neuron. PPC is a bias-free measurement of the phase synchronization of neuronal spiking in relation to LFP (Vinck et al., 2010). Our analyses suggested that the three PV interneuron subtypes show equal phase-locked firing with different oscillations, as the PPCs between interneuron subtypes did not show any significant difference (Kruskal-Wallis Test, *p*_4 Hz_ = 0.099, *p*_*theta*_ = 0.607, *p*_*slow gamma*_ = 0.691, *p*_*fast gamma*_ = 0.890, *p*_*HFOs*_ = 0.443; Median Test, *p*_4 Hz_ = 0.160, *p*_*theta*_ = 0.472, *p*_*slow gamma*_ = 0.558, *p*_*fast gamma*_ = 0.558, *p*_*HFOs*_ = 0.558). However, comparisons of PPCs between different frequency-bands showed that the rhythmic firing of PV interneurons occurred more robustly at high-frequency range rather than at other frequency ranges (Figures 3E, Friedman Test, *p* = 7.77e-9, Dunn-Bonferroni *post hoc*, *p*_4 Hz__–__*HFO*__*s*_ < 0.001, *p*_*theta*__–__*HFOs*_ < 0.001, *p*_*slow gamma*__–__*HFOs*_ < 0.001, *p*_*fast gamma*__–__*HFOs*_ = 0.016, *n* = 18 neurons). These results suggested that neural dynamics of PV interneurons are prominently related to, or modulated by, HFOs during AW state.

### Phase Coupling of PV Cells With Delta and HFOs During SWS State

Both cortical and hippocampal PV interneurons are mainly derived from medial ganglionic eminences (MGE) during development (Xu et al., 2004). The activities of PV interneurons in the hippocampus are strongly phase-locked to LFP sharp wave ripples (100–250 Hz) during SWS (Klausberger et al., 2003). We wonder whether similar features could be found between the firing of PFC PV interneurons and high frequency oscillations.

**FIGURE 4:**
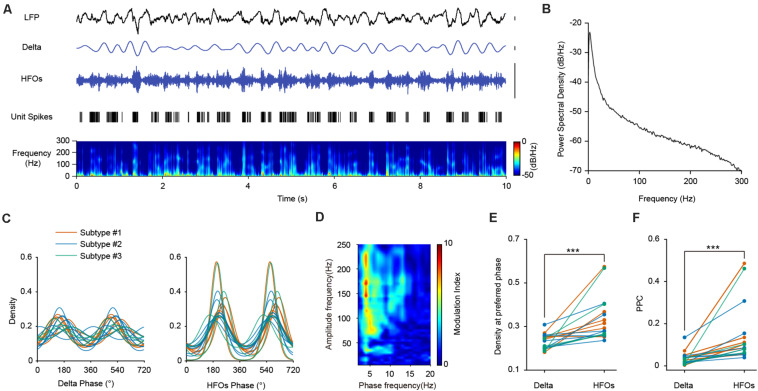
The firing phase-coupling of PFC PV interneurons to LFP HFOs during SWS state. **(A)** Example traces showing PV interneuron spikes and different LFP oscillation components during SWS. The illustrated PV interneuron exhibited correlated firing with HFO events. Traces from top to bottom, PFC LFP signal, delta oscillation, HFOs filtered from LFP, PV interneuron spiking activity (each stick represents one action potential fired by the neuron), and power spectrogram of LFP (10 s of data is shown, scale, 0.1 mV). Note the highly correlated activities of neuronal spiking and HFOs. **(B)** Power spectral density during SWS state of one example recording. **(C)** Firing phase distribution of 3 subtypes of PV interneuron spikes with delta and HFO during SWS. Each colored line represents one single neuron (*n* = 18 neurons). The firing activities of all the recorded interneurons are significantly phase-locked to both delta and HFOs. **(D)** Phase-amplitude coupling analysis of mPFC LFP during SWS. **(E)** Peak phase coupling density with different oscillations at preferred phase of PV interneuron spikes during SWS state (Wilcoxon Signed Ranks Test, ***, *p* = 3.27e-4, *n* = 18 neurons). **(F)** Comparison of PPC of PV interneuron spikes with delta and HFOs during SWS states (Wilcoxon Signed Ranks Test, ***, *p* = 1.96e-4, *n* = 18 neurons).

The LFP signals in the PFC during SWS were mainly composed of delta and high frequency oscillations (Figures 4A,B). We first investigated the relationship between PV interneuronal firings and delta rhythms. Population analysis revealed that all opto-tagged PV interneurons exhibited significant phase-locked firing to delta oscillations, with the mean preferred firing phase at 160.24°± 44.69° (Figure 4C, left panel). Furthermore, the three PV interneuron subtypes showed similar PPC levels with delta frequency (Kruskal-Wallis Test, *p* = 0.272; Median Test, Median = 0.031, *p* = 0.435).

We then turned to investigate the temporal dynamics of PV neuronal firing with HFOs. We found that the two signals exhibited high correlations, as the spiking of an example PV interneuron temporally coincides with HFOs during SWS (Figure 4A). All the recorded PV interneurons exhibited significant phase-locked firing to HFOs, with a mean preferred firing phase of 218.85°± 22.78° (Figure 4C, right panel). There is no significant difference between the PPCs of the three neuronal subtypes at high frequency (Kruskal-Wallis Test, *p* = 0.354; Median Test, Median = 0.085, *p* = 0.558). Neuronal phase-locked firing of PV interneurons was much stronger with HFOs than that with delta oscillations (Figure 4E, Wilcoxon Signed Ranks Test, *p* = 3.27e-4, *n* = 18 neurons). Comparisons of PPCs at different frequency also showed that the PPCs at high frequency were significantly higher than that at delta frequency (Figure 4F, Wilcoxon Signed Ranks Test, *p* = 1.96e-4, *n* = 18 neurons), indicating that the phase preference of PV interneurons to HFOs were much more robust than that to delta oscillations.

### Optogenetic Stimulation of PFC PV Interneurons Suppressed Pyramidal Neuron Activities

In order to investigate the role of PV interneurons in regulating cortical network dynamics, we employed optogenetic strategies to see whether neuronal activities and field potential oscillations were altered upon such manipulation. We noted that laser pulse stimulations of certain frequency typically triggered artificial increases across certain LFP frequencies. As a result, we decided to use sustained optogenetic stimulations to avoid such evoked phenomena.

**FIGURE 5:**
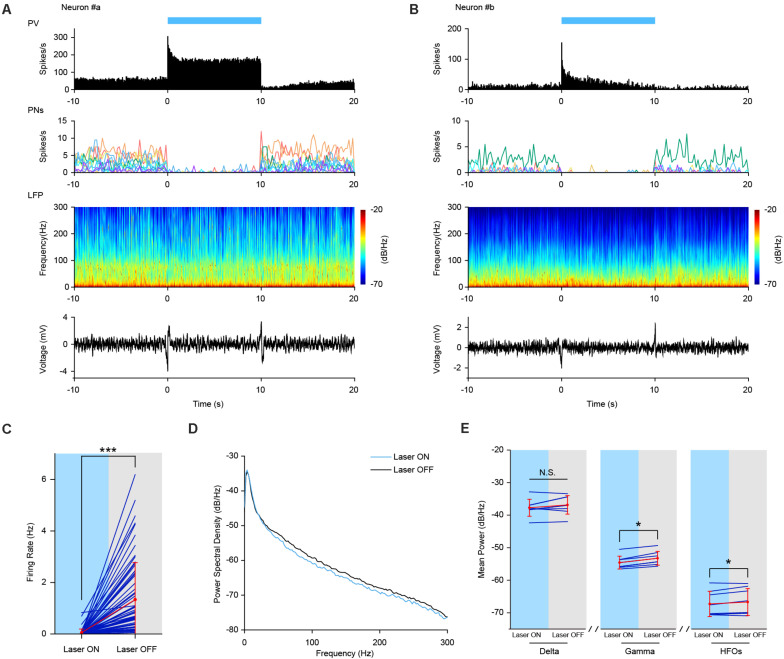
Increased Firing activities of PV interneurons by sustained optogenetic stimulation. **(A,B)** Neuronal firing activities of two example PV interneurons upon optogenetic stimulation in PV-ChR2-EYFP mice during AE and SWS state, respectively. Top row, neuronal firing responses of PV interneurons to sustained laser stimulation (10 s, bin = 0.05 s). Second row, neuronal firing activities of simultaneously recorded pyramidal neurons (PNs). Each colored line represents firing rate activities of one pyramidal neuron [*n* = 11 PNs in **(A)** and *n* = 7 PNs in **(B)**, bin = 0.2 s]. Third row, power spectrogram of PFC LFP. Bottom row, responses of PFC LFP to sustained laser stimulation. **(C)** Mean firing rate of simultaneously recorded prefrontal pyramidal neurons during sustained laser stimulation of PV interneurons. Red, mean ± SD; navy, individual; the same below (Wilcoxon Signed Ranks Test, ***, *p* = 3.5e-12, based on negative ranks, *n* = 65 neurons). **(D)** PSD of PFC LFP upon laser stimulation of PV interneurons during SWS state. Oscillation power at high frequency range (including gamma and high frequency oscillations) was slightly lower than that without stimulation. **(E)** Statistical test of mean power of different oscillations with and without sustained laser stimulation (Wilcoxon Signed Ranks Test, *p*_*delta*_ = 0.123, *, *p*_*gamma*_ = 0.012, *p*_*HFOs*_ = 0.049, *n* = 8 trials).

Figures 5A,B illustrated the laser induced firing responses of two representative PV interneurons to sustained optogenetic stimulations (470 nm laser, 10 s duration) during AW and SWS states, respectively. The PV neurons exhibited robust transient burst firing at the laser onset. After the initial burst, the laser evoked firing of PV neurons decreased to an elevated firing level (in comparison to the baseline firing rate). Firing rate during the stimulation period remained either stable elevation (example Neuron #a) or tampered off gradually (example Neuron #b). After the laser stimulation offset, the firing rate of PV neurons returned to baseline.

Next, we checked the effect of laser stimulation on the firing activities of local pyramidal neurons. As expected, the firing activities of all the simultaneously recorded pyramidal neurons were greatly suppressed during laser stimulation (see individual example cells in the second row in Figures 5A,B). At the neuronal population level, the mean firing rate of pyramidal neurons was significantly decreased from 1.34 ± 1.43 to 0.05 ± 0.14 Hz (Figure 5C, Wilcoxon Signed Ranks Test, Laser OFF-Laser ON, *n* = 65 neurons from 6 mice, *p* = 3.52e-12, *Z* = −6.955, based on negative ranks).

At the field potential level, PSD analysis revealed a slight decrease in the power of field potential oscillations, especially at higher frequency range during SWS state (Figure 5D). Statistical analysis confirmed that LFP power was significantly decreased at gamma and high frequency oscillation, but not at delta oscillation range upon laser stimulation (Figure 5E, Wilcoxon Signed Ranks Test, *p*_*delta*_ = 0.123, *p*_*gamma*_ = 0.012, *p*_*HFOs*_ = 0.049, *n* = 8 trials). The strong coupling of PV interneurons with LFP HFOs might contribute to the decreased oscillation power at high frequency range caused by prolonged optogenetic stimulation of PV interneurons in the PFC.

## Discussion

Combining Cre-mediated optogenetics with multi-channel tetrode recording, we were able to describe the *in vivo* firing patterns of mPFC PV interneurons in mice during four basic behavioral states. Most of the mPFC PV interneurons (14 out of 18) are modulated in a behavior state-dependent manner, often with elevated firing rates during AW and/or REM states in comparison to that during QW and SWS states. We identified three subtypes of PV interneurons based on their firing patterns: subtype #1 interneurons showed significant higher firing rate during AW than any other behavior states; subtype #2 interneurons showed elevated firing under AW and REM states compared to QW and SWS states; the firing rate of subtype #3 interneurons is stable across all behavioral states. Using parvalbumin as a molecular marker to categorize cortical interneurons only gives us a sketchy classification of interneuron types. In fact, our findings supported the fact that PV interneurons can be further categorized into subtypes based on morphological characteristics and detailed molecular expression profiles (Kepecs and Fishell, 2014; Zeisel et al., 2015).

Various studies have demonstrated that fast-spiking interneurons, especially PV positive interneurons, are crucial for the rhythmogenesis and function of mPFC gamma oscillations. Previous study in anesthetized rats has identified two sets of fast-spiking interneurons, firing at early and late phase of cortical UP states, respectively (Puig et al., 2008). Increased synchrony of fast-spiking interneurons at gamma frequency during cortical UP states is reported in both brain slice and anesthetized mice (Salkoff et al., 2015). Furthermore, computational studies predicted that PV interneurons play a crucial role in shaping cortical oscillations. Interconnected PV interneurons are expected to induce gamma oscillations (Buzsaki and Wang, 2012; Keeley et al., 2017). Dysfunction of PV interneurons shortened the duration of cortical UP state in PV-GAD67 mice (Kuki et al., 2015). Synchronized mPFC PV activities are characteristic during attention, accompanied by elevated power of slow gamma oscillation. The firing activities of mPFC PV interneurons are also phase-locked to slow gamma (30–40 Hz) oscillations during attention (Kim et al., 2016). Furthermore, optogenetic stimulation of mPFC PV interneurons at theta-gamma coupled frequency can positively modulate social behaviors (Cao et al., 2018). In our study, we investigated the *in vivo* firing pattern of single PV interneuron and their firing phase relationship with slow and fast gamma oscillation under natural behavior states (but not under attention or memory tasks). Consistent with previous reports, we found that some PV interneurons showed significant phase-locked firing with both slow and fast gamma oscillations. But the strength of such phase-locked firing with gamma oscillations are much weaker compared to that with cortical HFOs (Figures 3D,E). Further experiments would focus on unveiling the causal relationship between PV interneuronal firing and cortical gamma oscillations with optogenetic approaches. In our preliminary experiment, the sustained optogenetic activation of PV interneurons moderately decreased the power of gamma oscillation in the PFC during sleep. We speculate that cortical PV interneurons play a limited role in regulating gamma oscillations.

By applying phase analysis methods, we showed that PV cells in the PFC exhibited robust phase-locked firing to high-frequency oscillations (100–250 Hz) and delta rhythms (1–4 Hz), but poor coupling to gamma (30–80 Hz) or theta (4–8 Hz) oscillations. Moreover, PV interneurons change their firing pattern when behavior state altered, yet they always remain phase-locked to the troughs of high-frequency component of LFP. Ripple oscillations (100–250 Hz) in the hippocampal CA1 region reflect summed IPSPs in ensemble pyramidal neurons. They play an essential role in memory consolidation during sleep. Ripple oscillations always cooccur with large depolarizing activities, called sharp waves, forming sharp wave-ripple complex (Ylinen et al., 1995). The generation of sharp wave ripple events requires inhibitory inputs from local interneurons (Stark et al., 2014). Many hippocampal interneuron subtypes, including PV basket cells, have been reported to be coupled with sharp wave ripples (100–250 Hz) (Klausberger and Somogyi, 2008). Cortical high-frequency oscillations and hippocampal ripple oscillations share several common features: (1) They show overlaps in frequency band, both ranging from 100 to 250 Hz. (2) They are both prominent during SWS states (Buzsaki et al., 1992). (3) Hippocampal pyramidal ensemble burst out synchronous discharge at ripple troughs (Klausberger et al., 2003), while cortical pyramidal population exhibit synchronized spiking activities during HFO periods (Scheffer-Teixeira et al., 2013). (4) The coupling of PFC PV interneurons with high frequency oscillation bears a strong resemblance to that of hippocampal PV basket cells with ripple oscillations. These studies, together with our present results, suggest that PV interneurons in the hippocampus and PFC share good similarities in this regard. They may both involve in the generation and modulation of cortical and hippocampal high frequency oscillations (Tort et al., 2013).

All the simultaneously recorded pyramidal neurons (*n* = 65) showed significantly reduced firing rate during sustain laser stimulation (Figure 5C), confirming that PV interneurons in the PFC exhibit robust inhibition onto pyramidal cells. The prolonged stimulation of PV interneurons increased inhibitory driving force onto postsynaptic pyramidal neurons, hence reducing their firing activities as well as field potential oscillations. The optogenetic stimulation induced moderate effects on LFP power might also be resulted from the possibility of limited numbers of PV interneurons that were activated by laser stimulation through the optic fiber.

Technically speaking, our present study provides several useful perspectives in terms of optogenetic methods in the PFC. It is quite difficult for PV interneurons to follow a strand of laser stimulation at frequencies higher than 10 Hz, although some PV neurons were able to fire at a rate higher than 40 Hz under natural physiological conditions. Unlike physiological conditions, laser stimulation would induce a synchronized firing in PV population. Such synchronized activation of PV interneuron population may result in a strong temporal summation of inhibitory inputs. It may take tens of milliseconds for the membrane potential of PV interneurons to recover to the resting potential, consequently preventing PV interneurons from further adapting to the pace of the optogenetic stimulation frequency. This phenomenon of abundant inhibitory innervations between cortical PV interneurons had been reported by several previous studies as well (Anikeeva et al., 2011; Kvitsiani et al., 2013). Thus, such characterization argues that investigators should prefer to use stimulation frequencies lower than 10 Hz as a way to artificially enhance the function of PV interneurons.

In summary, we found that PV interneurons in the mouse PFC are consisted of at least three subclasses with distinct behavior state-dependent firing patterns. Moreover, activities of PFC PV interneurons were closely coupled to HFOs and delta band. PFC PV interneurons can readily regulate the firing of local pyramidal neurons, and high frequency components of the LFPs.

## Data Availability Statement

The raw data supporting the conclusions of this article will be made available by the authors, without undue reservation, to any qualified researcher.

## Ethics Statement

The animal study was reviewed and approved by the Laboratory Animal Management Committee at East China Normal University.

## Author Contributions

YY and LL conceived this project and designed the experiments. YY performed *in vivo* electrophysiological recording, immunohistochemistry experiments, and data analysis. LW helped with immunohistochemistry experiments and data analysis. YY and MW performed raw data processing. YY, LL, and JX wrote the manuscript. All authors contributed to the article and approved the submitted version.

## Conflict of Interest

The authors declare that the research was conducted in the absence of any commercial or financial relationships that could be construed as a potential conflict of interest.
